# Food and Beverage Advertising Aimed at Spanish Children Issued through Mobile Devices: A Study from a Social Marketing and Happiness Management Perspective

**DOI:** 10.3390/ijerph17145056

**Published:** 2020-07-14

**Authors:** Gloria Jiménez-Marín, Rodrigo Elías Zambrano, Araceli Galiano-Coronil, Rafael Ravina-Ripoll

**Affiliations:** 1 Audiovisual and Advertising Department, Faculty of Communication, University of Seville, 41012 Seville, Spain; gloria_jimenez@us.es (G.J.-M.); rodrigoelias@us.es (R.E.Z.); 2 Marketing and Communication Department, Faculty of Social Sciences and Communication, University of Cádiz, 11003 Cádiz, Spain; araceli.galiano@gm.uca.es; 3 Business Organization Department, Faculty of Economics and Business, University of Cádiz, 11003 Cádiz, Spain

**Keywords:** advertising, children, educommunication, food, happiness management, health, mobile devices, social marketing

## Abstract

Eating Disorders (ED) and obesity are a pandemic in developed and developing societies. In 2018, Spanish Ministry of Health and Consumption reported data on obesity (15%) and ED (12%). Spain thus ranks fifth among European countries in childhood obesity, with the highest incidence in the 6–12-year-age group. Many studies point to media as one of the contributing elements to this growth. In this sense, it should be noted that Spanish children are exposed to an average of 9000 television commercials per year and the vast majority of these are for food and beverage products of little or no nutritional value. Educommunication becomes essential here, since media have the capacity to educate, prevent and influence the behaviour as part of their social marketing strategies and within the happiness management philosophy. The aim of this paper is to analyse food and beverage advertising on mobile devices aimed at children. The methodology used includes a content analysis, a survey, and focus groups. The results show that many of the food products are bought or ordered as a direct result of advertising. The main conclusions point to the need to regulate the messages transmitted in order to guide the social function of media so that public health and happiness can be improved.

## 1. Introduction

This article analyses food and beverage advertising on mobile devices aimed at children. This study found that many of the food products are bought or ordered as a direct result of this advertising.

### 1.1. About Children’s Media Consumption

The impact of mobile devices on Internet consumption is a reality that affects all countries and all age groups. In the Spanish case, 91.4% of households had Internet access and 66.0% of the population aged 10 to 15 years old had a mobile phone, and the older they are, the greater the use of ICTs, especially from the age of 13 years old onwards [1].

Mobile devices are the main gateway to Internet access for children. Similarly, features such as the portability, immediacy or ubiquity of these devices, generate more barriers to parental mediation [2].

Based on Reception Theory, some studies [3,4,5,6,7,8,9] have demonstrated the growing interest in the study of communication aimed at children. In the same sense, Cultivation Theory [10] assumes that individuals develop beliefs, attitudes and expectations about the real world based on what they see and hear in media and then use them to make decisions. This is particularly true for children’s audiences, since children’s programming uses strong emotional connections and children build their identities within the consumer culture and media in which they are immersed, being consumers from a very early age [11].

The literature considered eight years to be the age at which the child was aware of the persuasive intent of advertising [12]. A child was also considered to have acquired the skills and performance of an adult consumer at that time [13]. However, other studies that focused on children in this age group showed that they do not perceive the persuasive vocation of the advertisement and only process the playful and entertaining part of the content [14]. In this respect, some authors consider that when children consume advertising new media, they are exposed to certain risks of which parents are less aware, and, as a result, parents have more difficulty in perceiving and controlling this persuasive communication [15]. Besides, dealing with advertising in a critical way requires the development of cognitive skills, which are given as the child grows [16]. Therefore, following the Processing of Commercialized Media Content (PCMC) model [17], it can be argued that children apply low-effort cognitive processing when faced with advertising and they do not activate the knowledge associative network they have developed about the phenomenon. Thus, it can be said that children are the social group most sensitive to the negative consequences of the media, assuming, to a large extent, dependence on the consumer market [18]. Consequently, some studies [19,20,21] consider that children have fewer cognitive resources to correctly decode the messages received by the media.

### 1.2. About the Influence of the Media on Food-Related Diseases

There is a great deal of scientific evidence that points to a close relationship between children’s exposure to the media and these diseases [22,23], pointing to different factors that would explain this association [24,25,26,27,28]. However, the role of advertising, and specifically that of ultra-processed and unhealthy foods, is emphasized as the most relevant [29], because the greater credulity and lesser experience of children [30,31] the more susceptible they are to being influenced by the persuasive strategies used in commercial communication [32]. Because advertising can generate representations that involve image patterns and unhealthy behaviours [33], and this can directly disturb children’s self-esteem and, with it, the construction of their own body image, resulting in a high dissatisfaction of children regarding their bodies and thus producing physical and psychological disorders. This capacity of influence can already be seen from studies [34,35,36,37,38,39,40,41] which show that half of children between 5 and 12 years of age are dissatisfied with their physical appearance [42,43]. In the same line, other studies states that around 40–50% of children aged 6–12 are dissatisfied with their physical appearance [44,45,46].

While recent research on the eating disorder has reported stability in the incidence of anorexia and decreased bulimia [47,48], there is a notable increase in the obesity levels of children under 12. The Ministry of Health and Consumer Affairs has been providing data since 2006 and the World Health Organization (WHO) [49] estimates that child obesity is around 14%, indicating that overweight is over 12% and it also coined in 2001 the term “Globesity” to define the severity of the obesity pandemic in the world. Estimates of the prevalence of obesity and/or overweight in the countries of the Organization for Economic Cooperation and Development (OECD) and other emerging countries in the child population between 5–17 years provide average values of around 22% [50].

The data found in several studies [51] raise questions such as the fact that child obesity has tripled in Spain, occupying second place among European countries in terms of the prevalence of overweight among children between 6 and 12 years. This observation is confirmed when we look at data for the year 2020 [52]. However, a reduction in overweight is expected provided that the recommendations of some studies are followed [53].

In this sense, several authors [54,55,56] refer to disorder in relation to the perception and real messages understanding that children receive through these apps and platforms, due in part to the lack of regulations in this regard. 

Thus, the European Union’s concern for the protection of children in the audiovisual sector has materialized in a series of initiatives that have led Spain, among others, to develop more specific regulations for minors. In the area of regulation of food and beverage advertising aimed at children, the Spanish General Law on Advertising (LGP) [57], the Spanish General Law on Audiovisual Communication (LGCA) (Law No. 7, 2010) [58] or the European Law on Food Safety and Nutrition (Law No. 17, 2011) [56] are noteworthy. The European Food Safety Authority (EFSA) [59] is an independent body responsible for advising governments on the existence of food risks [60]. In the case of Spain, it has the coregulatory attention of the Spanish Agency for Consumer Affairs, Food Safety and Nutrition (AECOSAN) [61] whose main tool is the Code for the Regulation of Food and Beverage Advertising to Children, Prevention of Obesity and Health (PAOS Code) [62], applicable to media such as television and, since 2012, Internet (including mobile devices). This code establishes a set of ethical norms that must guide member companies in the development, execution and dissemination of their food and beverage advertising messages to minors to avoid excessive advertising pressure on them. 

However, regulations aimed at prevention are quite limited, as there is some regulation, but they are relaxed in relation to compliance with their codes [63], or insufficient [64].

This is where social marketing comes in, trying to influence people’s behaviour in search of a more beneficial target [65]. In this sense, researchers [66] propose strategies for organizations to try to understand the use of social media and for organizations to determine how they transform their supporters into vocal promoters of their causes [67]. Some research has also shown that social marketing can go beyond awareness raising and behaviour change by mobilizing its target audience [68,69,70], as happiness and the achievement of well-being remains an identifiable goal pursued by private companies and public institutions [71], as well as advertisers, EFSA [59], WHO [49] and consumers (children and parents).

## 2. Research Objectives

This research is based on three premises: firstly, that in order to develop a critical attitude towards advertising, the child must first of all be able to perceive the phenomenon [72], which does not usually happen at an early age; secondly, that when referring to content (and advertising) broadcast on mobile devices, parental control is loose; and, thirdly, that food and beverage advertisers are taking advantage of these two facts to impact the receiving public (consumer and prescriber), in many cases ignoring the recommendations of the WHO [49] and the PAOS Code [62]. Hence, an attempt will be made to answer the following research questions:

- Do children identify the advertising content to which they are exposed while consuming the Internet via mobile phones? Do they trust them?
- Do food and drink advertisers follow the norms of the PAOS Code?

The aim of this research is to study the advertising of food to children on mobile devices, before and after the adoption of the code, and thus:

(1) To find out if children identify the advertising content to which they are exposed as such.
(2) To contrast the advertising rigor of the PAOS code with the agency’s advertising actions within the Spanish area of action for its compliance.
(3) To extract a list of the main food product advertisers that approach children under 12 through apps, interactive games or advertising in traditional formats.
(4) To evaluate the existence of professional ethics in this advertising sector.

## 3. Materials and Methods 

### 3.1. Material Design

**Figure 1:**
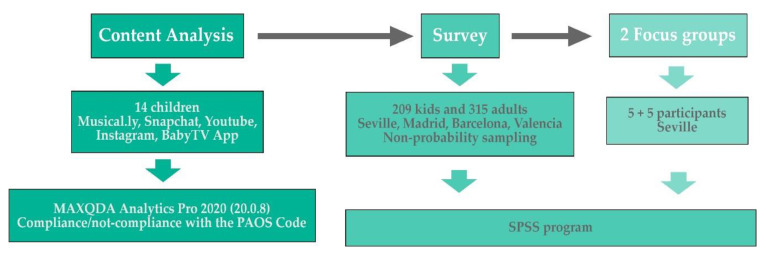
Used methodology.

The methodology used is mixed. By way of illustration, the development of the methodology used can be seen in Figure 1:

#### 3.1.1. Content Analysis

First, we used a content analysis where the sample was obtained through the recording for 7 days (week of 19–25 August 2019) of the advertising content issued by the advertisers, inserted in content aimed at the child audience.

Although there is currently a protected television schedule (from 6:00 to 22:00), this does not apply to advertising on mobile devices, who’s parental control must be activated by the parents or children’s carers. It is for this reason that the recordings took place at different times throughout the day, including between 10:00 and 21:00. 

The analysis method was based, on one side, on a content analysis of food and beverage advertising aimed at children under 12 years old on mobile devices in Spain. Specifically, we analysed advertising directed at kids through Musically, Snapchat, YouTube, Instagram and BabyTV App. The criteria for choosing these applications and pages was basically the audience [70]. 

Regarding the advertising inserted in the selected apps, 14 children, all urban residents, were recorded for a full week each time they used the mobile devices. The 14 children were sampled by non-probability and non-random convenience sampling, to create samples according to ease of access. Members were chosen after selecting the age range and sex. All were chosen thanks to data provided by several primary schools in the four selected Spanish cities (Seville, Madrid, Barcelona and Valencia). Each child was assigned to only one application, which was repeated in two cases. In this way, each application was on the device (mobile, tablet) of two children. Each time the child (or his or her parent) decided to use the device, it had to be done in a specific location in the child’s home where a camera was set up to record, externally, the content that appeared and the child’s interaction. Due to the data protection policy, these recordings are only available to researchers.

The aim of this content analysis was basically to detect the main advertisers and brands that frequently use advertising in these applications, websites or social networks. 

The ads were classified according to their compliance with the PAOS Code and its ethical standards at three different levels: (a) compliant; (b) non-compliant; and (c) unclear [62]. Using this classification and based on their visualization by the researchers and the data was tabulated. Compliance with each standard was evaluated considering that they were in full compliance with the PAOS Code [60] when none of the requirements or standards were circumvented, i.e., they were considered non-conforming if some of the internal standards to each standard were not met. Those whose compliance was lacking consensus by external and impartial investigators and experts were considered as ‘unclear compliance’. Moreover, for content analysis we use the analysis tool MAXQDA Analytics Pro 2020 (20.0.8).

#### 3.1.2. Survey

In addition, at the quantitative level, a survey was conducted. A total of 524 persons (children between 5 and 12, and their mothers, fathers or guardians) were interviewed. Specifically: 209 kids and 315 adults. This was achieved thanks to the Google Forms tool. All of them were urban habitat residents.

The participants of this part of the study (the survey) were Spanish children between 5 and 12. They were selected for research using non-probability sampling, which included the following steps. First, potential participants were reached through contacts with different schools in several Spanish cities (Seville, Madrid, Barcelona and Valencia). This choice was made in order to obtain maximum representativeness, although we must point out the limitations of this research, as we found it difficult to extract a large sample representing the entire national territory.

To guarantee the confidentiality of the research and ensure that the children belonged to the sample of interest, they were administered, through their parents, a filtering questionnaire to identify age characteristics, in addition to the fact of having both an Internet connection and a mobile device where he could access the content. Only those children who met the characteristics required for the study were invited to participate in this part of the study.

#### 3.1.3. Focus Group

Furthermore, two focus groups were held. The participants were 10 people: eight mothers and two fathers. For logistical reasons it was held only in the city of Seville (so, all of them were urban residents). The parents of 10 different children from two different schools in the same city were chosen for the demonstration. To meet the objectives of the study, two focus groups were established with the same profile of participants, although they were held at different times (26 August 2019 for the first; 2 September 2019 for the second). Each group had five participants (four women and one man in each case).

For the preparation of this article, a univariate and bivariate statistical analysis has been applied to the data set collected with the help of the SPSS software (version 25, IBM SPSS Statistics, Endicott, NY, USA).

In this way, it has been possible to study a whole series of variables in relation to such content: products advertised, compliance or not with the PAOS Code, and children and their parents’ perception.

The points that safeguard the safety of children and adolescents within the PAOS code [60] and, therefore, its ethical standards in relation to food regulation in advertising are:

- Principle of legality: Advertising for food or beverages shall comply with current legislation.
- Principle of loyalty: Advertising for food or beverages shall conform to good faith and good business practices.
- Education and nutritional information: Bad habits and sedentary lifestyles shall not be promoted, but healthy habits and the maintenance of a varied diet shall be promoted.
- Presentation of the products: The products promoted must not mislead or generate confusion in children, must not lie about their benefits and must be realistic without exploiting their imagination with special effects or that the toys move by themselves.
- Product information: The product or information about it should present the characteristics in a clear and simplified manner to facilitate understanding.
- Sales pressure: Advertising will not exploit children’s inexperience and disbelief, nor will it encourage persuasion from parents or legal guardians. The benefits of the product should be inherent in its use and finally they should not be pressured into buying it.
- Support and promotion through characters and programs: Advertising will not exploit children’s confidence in such a way as to use teachers, family members or charismatic characters for them, which attract their attention.
- Identification of advertising: Advertisements aimed at children under 12 should be clearly separated from programmes.
- Comparative presentations: Sometimes advertising makes comparisons to demonstrate certain characteristics, and these comparisons must be clearly understandable to children.
- Promotions, sweepstakes, contests and children’s clubs: These should not raise unrealistic expectations about the chances of winning the contest or the prize that can be won.
- Safety: Advertisements should avoid violent scenes that encourage misuse of the product. They should not encourage the child to enter strange places or interact with strangers.
- Treatment of personal data: It must be formalities to recognize these children and in no way, they can be used outside the exchange of product-services. In the online environment, the formalities will be used to facilitate procedures for access, rectification, cancellation.
- Viral marketing: Companies that carry out viral marketing campaigns will not capture data from third party recipients.
- Protection against inappropriate content: Companies in charge of advertising must not display inappropriate content on their portals or associated websites, such as improper advertising. In their aforementioned areas or zones selected for kids, there must not be any content that causes any harm, either physical or mental, to the children.
- Within each of these norms, there are a total of 32 standards that specify the aspects that are regulated by this self-regulation code.

Similarly, it should be noted that this code considered ethical standards established at European and international level on the advertising of food and beverages. The most relevant standards on which this code is based are:

- Law 34/1988, of 11November, General of Advertising.
- Law 3/1991, of 10 January, on Unfair Competition.
- Royal Legislative Decree 1/2007, of 16 November, approving the revised text of the General Law for the Defense of Consumers and Users and other complementary laws.
- Royal Decree 1334/1999, of 31 July, which approves the General Rules on the Labelling, Presentation and Advertising of Food Products.
- Royal Decree 1907/1996, of 2 August, on advertising and commercial promotion of products, activities or services with a supposed health purpose.
- Law 7/2010, of 31 March, General of Audiovisual Communication.
- Organic Law 15/1999, of 13 December on the Protection of Personal Data.
- Royal Decree 1720/2007, of 21 December, which approves the regulations implementing Organic Law 15/1999, of 13 December, on the Protection of Personal Data.
- Law 17/2011, of 5 July, on Food Safety and Nutrition.
- Regulation 1169/2011 of the European Parliament and of the Council on food information provided to consumers.
- Regulation 1924/2006 of the European Parliament and of the Council of 20 December 2006 on nutrition and health claims made on foods. European Parliament resolution of 15 December 2010 on the effects of advertising on consumer behaviour.

### 3.2. Procedure and Data Analysis

On the one hand, and by means of the quantitative methodology, this work counted the advertisements that have appeared related to the object of study; on the other hand, and for the qualitative analysis, the research is supported by the content analysis, so that the compliance/non-compliance/of the advertising pieces can be identified according to the different terms that are used in them and how they relate to each other, an identification in which MAXQDA2020 will help. This program allows us to export clouds and word combinations in jpg., as well as data in Excel format. Both formats are combined in this work. Of the total of 314 ads, 73 were analysed for food and beverage, thus n_1_ = 73.

The survey includes a total of 16 items analysed through statistical processing. Before applying the final survey on the sample, a pilot pre-test is carried out with 10 questionnaires. Initial difficulties are detected, and therefore some of the results were invalid. The questionnaire was therefore rewritten, with questions of a different nature or reformulated to obtain greater and more specific information. Subsequently, we moved on to a sample of 20 and, based on the suggestions received, the model questionnaire was closed. With the feedback received, the method is definitively implemented.

The program chosen for the survey is IBM SPSS Statistics (version 25, Endicott, NY, USA). The margin of error is calculated with the Scott Pi formula, reaching a reliability level of 0.98. A total of 524 responses (n_2_ = 524) was therefore analysed.

Transcripts of each discussion group were made from the notes from the observers (the researchers) and using the video and audio recordings as support. To analyse these transcripts, a content analysis was conducted by following the steps below:

(1) First, each discussion was divided into text fragments related to the main topics and established in the guide, previously elaborated.
(2) In each excerpt, each of the individual observers highlighted the key words representing the main ideas or logical groups of information.
(3) The keywords were then grouped into categories by each of the observers, also separately.
(4) The categories were compared among the observers to identify similarities. If there was enough agreement, these categories and their meaning (inferred from the text fragments) were restated in statements. If there was not enough agreement, a consensus could be seen among observers on the exact meaning of the issues reached during the session.

As mentioned above, the responses of the 10 people who participated in two similar focus groups of five people have been analysed. Therefore n_3_ = 10.

## 4. Results

### 4.1. Results of the Content Analysis

During the 7 days that the sample collection took place (11 h a day), a total of 77 h was counted where a total of 314 advertisements were found directly addressed to children. Of these, 73 were advertisements for food or drink.

Of the 73 advertisements that made up the total sample directly related to the object of study; the total number of advertisers was 22. Thus, the brands responsible for this advertising were, in order of appearance (original Spanish brands): (1) Ositos; (2) Babybel; (3) Fanta; (4) Danone; (5) Danonino; (6) La vaca que ríe; (7) Galletas Príncipe; (8) Chips Ahoy; (9) Choco Flakes; (10) Haribo; (11) (caffeinefree) Coke; (12) Danup; (13) Telepizza; (14) Hero baby; (15) McDonalds; (16) Donuts; (17) Burger King; (18) Tosta Rica; (19) Puleva; (20) Oceanix; (21) Dinosaurus; and (22) Sunny Delight.

Table 1 (PAOS Code compliance and its ethical norms in mobile devices in Spain, 2019) shows the level of compliance with each ethical norm of the PAOS Code. The ethical norm that shows the highest rate of non-compliance with the code is principle number V (Information about the products). Only 6.84% are in compliance with norm IX, language understandable to this public, in a clear, legible and outstanding way. Careful overprints in size and time of permanence were not suitable, using terms and texts unconnected with this public and hence leaving the mere recreation of visual stimuli as the attention-grabbing factor by inciting impulsive consumption. Another ethical norm with a lower rate of compliance with the code is IV. Presentation of the products, given that there is continuous non-compliance, with only 12.33% of the commercial pieces complying with the standards. Specifically, four of the standards in norm IV stand out:

- Standard 4. Adoption of special caution in the making and dissemination of food and beverage advertisements (represented by their Spanish acronym AAB) to children under 12 years of age in order to ensure that presentations do not induce errors about the product.
- Standard 5. Not to induce errors about the benefits derived from the use of the product.
- Standard 6. Should not mislead by suggesting that the food product being promoted has characteristics to other products in the sector.
- Standard 7. Precautions should be taken not to exploit the imagination of children under 12 by avoiding the use of fantasy elements, such as cartoons or animations, and the generation of unattainable expectations that exploit their vulnerability and innocence.

On the other hand, it is not only surprising that the level of compliance with the previous norms is low, since there are high percentages of uncertainty (unclear) in their compliance with rates of 34.25% and 38.36% respectively, in the order described above, but also the rates of non-conformity of norms X and XI, with the former standing out to a greater extent. Ethical norm X. Promotions, raffles, contests and children’s clubs, with 53.42% of non-compliance with the code violates the following standards on a recurring basis:

- Standard 18. The advertising message including a promotion should be designed to clearly show the advertised product and not just the promotional item. In that respect, as regards whether the principle of legality is complied with, there are two different versions of one advertising piece, DanUp, which are broadcast almost alternatively, and which is not in conformity with and uncertain compliance with that norm. It does not fully comply with the contract linking it to the incipient 2020 Plan for Supporting Base Sports due to the absence of the logo for less than 50% of the duration of the spot.
- Standard 19. The essential conditions of the promotional offers must be expressed in the advertising with simplicity and clarity.
- Standard 20. Advertising draws included in the F&B must not create unrealistic expectations about the chances of winning or about the prize that can be won. Therefore: Prizes must be clearly indicated; misleading about the “chances” of winning must be avoided; prizes awarded must be appropriate for this audience.
- Standard 21. In order to avoid misleading, reference to children’s clubs in the F&B may only be made if three conditions are met: Interactivity, continuity and/or exclusivity.

In the case of standard XI. Security, the non-conformity rate shows us a percentage of 42.46%, suffering a violation of standards 22 and 23, which indicate the avoidance of scenes, images or messages that encourage dangerous use of the product, or the incitement to enter strange places or talk to strangers.

Of the 32 ethical standards included in the 14 ethical norms that promulgate the PAOS Code, whose adherence is voluntary for companies in the food and beverage sector, each of these is continually violated. For this reason, Table 2 (shows the recurrent non-compliance with standards in the 177 advertisements, divided into 11 categories of products, which coincide with 11 different pieces of advertising whose frequency throughout the period analysed is shown in the table using the nomenclature: (a) The standards that show the highest percentages are standard 4 and standard 7. Regarding standard 4, which refers to special caution in the production and dissemination of advertising aimed at children in order to preserve their vulnerability and induce errors by the presentations of the product, there is a66.7% of non-compliance among the spots that do not comply with ethical standard IV (Presentation of products). Likewise, in relation to standard 7, which refers to the duty to take precautions against the exploitation of imagination using fantasy and the consequent generation of unattainable expectations, the percentage of non-compliance is the same. Both standards, included in the fourth norm, are also immersed in the advertising piece number 6, which presents greater incompatibility with the PAOS Code and which corresponds to the McDonald’s spot (with 10 of the standards not being complied and with more than 45% of the standards being infringed). Within this advertising there are also two of the standards with the highest rate of non-conformity, such as standard 12 (which refers to the use of benefits attributed to the product, given that these must be inherent to the product itself or its use), whose rate of non-conformity is 80% with respect to the standard. Finally, standard 19 corresponding to norm X. Promotions, raffles, contests and children’s clubs and it aims to ensure clarity and simplicity to make legible and understandable the special conditions of promotional offers; in this case, this standard is violated 100% of the time that the rule number 10 is violated.

Of the 32 standards included in the PAOS Code and evaluated individually, there is one that is even more violated: standard 9 of normV (Product information), which indicates the obligation to use understandable language in order to be in accordance with its public. It was violated in fiveof the 11 pieces and it was not complied, with 45.5% being the highest percentage of times not respected by agencies and advertisers. It should be noted that, of the 11 pieces, there were also 5 (45.5%) that were of uncertain compliance and where the consensus was minimal to be approved as consistent with the code.

**Figure 2:**
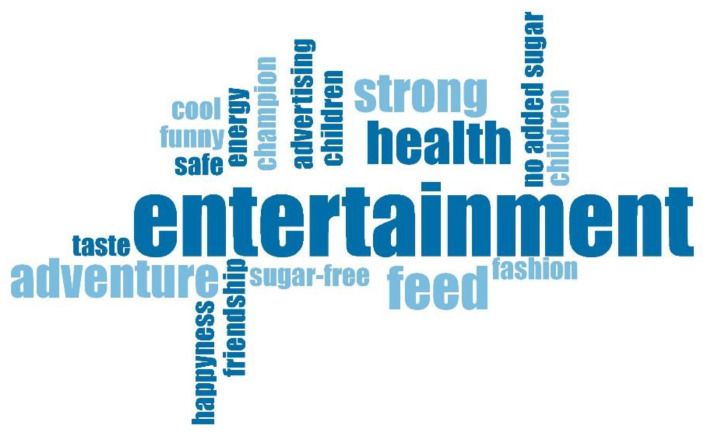
Words combinations found in the ads.

Overall, evaluating the level of compliance with the PAOS code, and although we represent it graphically in the following table, the summary is very basic: None of these brands meet the PAOS code 100%. This is shown in Table 3:

Within this analysis of content carried out, with the simple purpose of reinforcing the compliance/non-compliance/analysis of the advertising pieces, and using the results that the clouds of MAXQDA2020 have contributed, the term ‘entertainment’ is highlighted as the most frequent among the words with the most presence in the advertising. It appeared in 52.05% of the pieces. Above this word, we found in Figure 2 the term ‘entertainment’, appearing in 50.68%, followed by ‘health’, appearing in 36.99% and immediately followed by ‘strong’ and ‘adventure’, with a frequency of appearance of 19.20% and 18.67%, respectively.

### 4.2. Results of the Survey

A brief survey was carried out to determine the level of memory and recognition [73] of brands, products and advertising content, in order to analyse the possible advertising effectiveness. In this sense, the children surveyed were asked if they had visited any of the referred social networks, applications or portals (Musically, Snapchat, YouTube, Instagram and BabyTV App) in the last 7 days. Of the total of 617 people who responded to the survey, 524 had seen one of the marked applications and, consequently, the advertising inserted in them.

The survey was answered by 209 children under 12 and 315 adults, all parents. Among the most relevant data, the following stand out:

- The 209 children and 315 adults perfectly recognized the advertising brands when they were shown the packaging, or the brand was mentioned. Thus, when asked the question “Do you know which product this package corresponds to?”, all the respondents, children and adults, recognized the product when they saw the stimulus on the package.
- Of the total number of surveys, 52.01% remembered the brand or product without any previous external stimulus. In particular, the distribution was as follows:
  ⋅ 73 of the 209 children (34.92% of children).
  ⋅ 53 of the 315 adults (17.09% of the adults).

**Figure 3:**
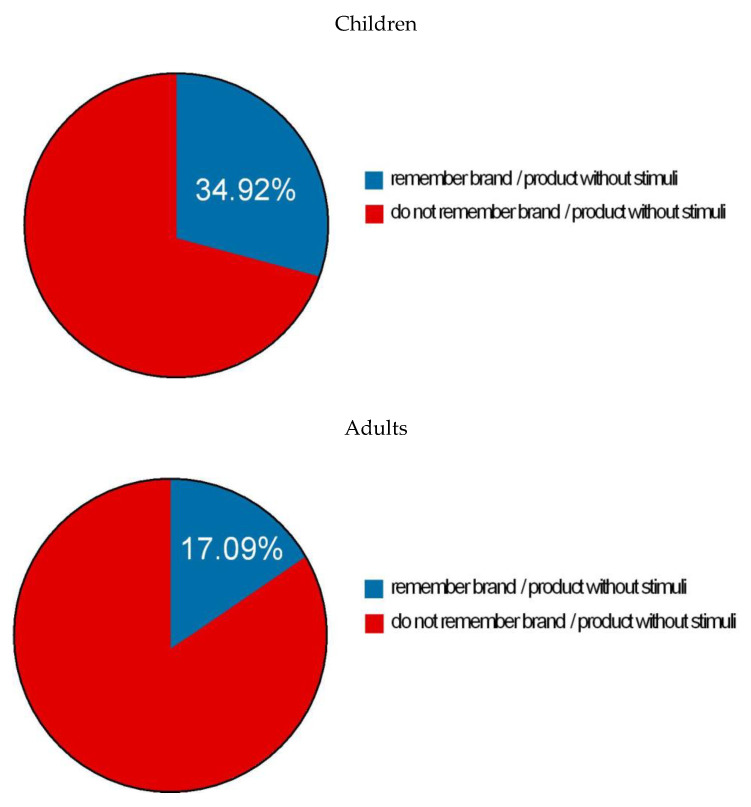
What product or brand do you remember watching advertised on the mobile apps you used during the last seven days?

This can be seen in Figure 3:

- In relation to the purchase intention, 100% of the 209 kids stated their desire to eat or drink any of the foods or beverages appearing in the advertisement shown; however, when the same question is answered by the parents, when the percentage of adults with no intention of buying or consuming any of the products shown is only 4.44% (14 adults). This may be logical to the extent that the level of training is much higher; however, what is striking about the data is that, apart from these 14 respondents, 198 (62.85%) made some of the categories coded as ‘uncertain’ as the first option of purchase, consumption or ingestion, as opposed to those coded as ‘non-conforming’.
- The brands best valued by parents are, in order:
  (1) Puleva;
  (2) Danone;
  (3) Danonino;
  (4) Danup;
  (5) La vaca que ríe;
  (6) MiniBabybel.

### 4.3. Results of Focus Group

In relation to the amount of time children spend in front of the media, the 8 people on the table admitted that children spend too much time in front of technologies; however, when asked to quantify how long they thought it was appropriate for children to be in front of a tablet or smartphone, responses varied considerably. Thus, compared to “*10 min a day*”, in the case of R. O. J., mother of two girls of 7 and 9 years old, we found the “*2 h at the most*”, which was answered by the father of two children, a girl of 5 and a boy of 6. Between these bands, intermediate answers were given that bet more on a moderation “*without counting exactly the minutes*”, and “*being aware that the exposure to mobiles is there and we cannot avoid it*”. Or, answers like “*I don’t get along very much and I don’t like it, but a little bit a day to let me breathe doesn’t hurt either of us*”.

In any case, we establish one of the focal points of importance of all the answers in the fact that the parents, when referring to the time that the kids are with a device, they do it in solitude, and not accompanied by a parent.

In this sense, and referring to the next block, all the participants in the focus were aware that the children were receiving advertising impacts but, in all cases, it was thought that the impacts received were reduced. In this way, when it was stated that during one week an average of more than 300 hits was received, some of the immediate comments were in line with “*from now on I won’t lend them my mobile phone*” or “*I had no idea of such an amount*”. But, in addition to this, knowing that 73 advertisements were directly influencing their children’s diet, the reactions were diverse: Thus, seven of the people in the focus group agreed that it was normal and that it was not food that was especially harmful to health, with some specific incidence: “*Coca Cola is not very good, but without caffeine, drinking it once a week I don’t think it’s very bad*”. Or “*yogurts are very good; it has always been said*”. The truth is that only three of the participants questioned the ingredients, the amounts of sugars, colorants, and preservatives, as well as the concept of healthy eating as a basis.

As a conclusion to the focus, four of the participants admitted that they bought many food products because of the pressures and incitements of their children, even when they did not agree. And even one person stated: “*Once you can refuse, and again, and again. But when the cookie comes with Paw Patrol, and you tell them you won’t buy it for breakfast. Besides, he doesn’t even want to see the fruit and between going to school with an empty stomach, or going to school with the cookies, then the Paw Patrol cookie is better!*”.

In addition, it is very noteworthy that in all the 10 cases the parents admitted that they bought many of the food products (either the original brand or a substitute brand) motivated by the advertising, or due to the express request of their children who, on many occasions, “*of course, see it in advertising and then ask you*”. Therefore, the prescription that the kids make is fundamental here, exercising their role of consumers and urging their parents to exercise the residual, but not negligible, role of buyers.

## 5. Discussion

There is a certain predisposition prior to the adhesion and linkage to the PAOS Code by the companies. However, this predisposition does not correspond with the purpose pursued by the administrations and the real achievement of the objectives, since it is clear that self-regulation is not an effective mechanism to avoid or reduce the exposure of children to advertising of unhealthy foods and beverages and, therefore, to promote a healthy lifestyle from childhood, as previous studies have evidenced [21,28,30,31,52,64] referring to other media, such as television. In this case, we study specifically mobile devices, because these previous studies did not look at other media different from television, and we are starting from the same base.

Although media education has become essential in a society in which children have, increasingly, access to different media and they are more exposed to them, the truth is that it must be taken into account that children under 12 have not yet developed the cognitive skills and critical capacity to discern or disagree with advertising and the social value that children give to the products and brands that are advertised [2,13,14,15].

The PAOS Code [62] sets out the regulatory framework for the design of food and drink advertising aimed at children. We note that the implementation of the PAOS Code [62] has not had much impact on the quantity and quality of food advertisements for children. In order to be effective in promoting healthy living, this regulation must be extended to all products and brands (healthy or unhealthy) that are likely to be perceived by children and that children may consume despite not being specifically aimed at them. Therefore, it should be remembered that, given its conditions, advertising is an area for the development of social communication that constitutes a challenge for institutions seeking to self-regulate it.

By understanding that most children remember and recognize brands and products from unhealthy food and drink advertisements, advertising is not self-regulating. For this reason, and due to the weakness with which the adhering companies are following the standards of the code, the existing regulatory framework must be revised as it is clearly ineffective. This research shows the need for stricter regulations on food and beverage advertising that can be perceived by children, even if it is not directly addressed to them. The results presented here have immediate implications, since we must ensure that the existing legal framework in Spain on the regulation of advertising is respected by food and beverage companies, making effective the legislation on health.

In accordance with other studies [52,53,64,65], it was found that among the most used strategies to advertise food are promotions or offers, the appearance of known characters, or giving them away as gifts, clearly violating some of the standards that appear expressly in the norm. According to what was observed, these advertising strategies could be classified as misleading or abusive since the food advertised is linked with properties that are actually difficult to find. Such methods of persuasion are misunderstood by children and this fact puts them in a clear disadvantage regarding the development of their preferences and choices.

To try to prevent health problems with serious implications at various levels, as childhood obesity, diabetes or psychosocial diseases such as anorexia or bulimia nervosa, we start from the need of being punished if the advertising companies does not meet the standards of the PAOS. They should pay fines high enough so that repeating the violation strategy is not an option.

In fact, as an approach and as a consequence of the data obtained, the need to amend the legislation may be considered in the following terms: (1) To prohibit the advertising of energy-dense foods in children’s programmes and media spaces; (2) To ban promotions and competitions for unhealthy food and drink for children; (3) Regarding advertising foods with low or no nutritional value, mentioning the possible side effects of their excessive consumption, both in advertising and on the product itself (as is already the case with tobacco in Spain) should be mandatory; and (4) Advertising for children should be much more limited in terms of broadcasting times and/or number of pieces advertised.

This paper has some limitations. First, the results are based only on the advertising of broadcasted through seven mobile device applications aimed at the Spanish audience, so it is not known if the same situation occurs in other apps or in other countries. Similarly, it is limited to the mobile audience and it does not reach the traditional media audience, such as television, although there are recent studies that already address this issue.

Secondly, the study was carried out in different geographical locations, such as Seville, Madrid, Valencia or Barcelona; it would be interesting to extend the study to other areas such as Extremadura or Galicia, to complete a map in this sense. However, despite these limitations, there are no other studies in Spain to the date that analyse the advertising to which Spanish children and adolescents are exposed through apps on mobile devices.

## 6. Conclusions

This study showed that children are consuming, with undesirable strategies, advertisements for foods, particularly sweetened drinks, sweets and cereals with added sugar, or carbohydrates, which are higher in calories, fats and sugars and have little or no nutritional value. In addition, advertising strategies such as associating the product with positive emotions and promotions are more frequently used in food commercials broadcast during children’s programmes. These findings can be considered when developing effective regulation of advertising to children and adolescents.

In order to evaluate the effectiveness of the PAOS Code in Spain, specifically in advertising aimed at children and broadcast on mobile devices, 73 ads were analysed and parents and children who have been confronted with these advertising pieces were interviewed. From the analysis carried out, it was concluded that the PAOS Code is not rigorously applied in Spain and that, on the one hand, companies seem to be unaware of it or violate voluntarily. On the other hand, health authorities and public administrations in charge of this control have the same problem: either they are unaware of this violation or they are not interested in censoring, a priori, such advertising. Even though the legal framework for food and beverage advertising in Spain is based on superior Community regulations, the truth is that, as already indicated by other researchers [52,64], it has serious deficiencies. As a support for this, our results show that:

(1) The same companies that voluntarily adhere to the NAOS strategy are those that violate the code of ethics.
(2) There is a high exposure of children under twelve to food and beverage advertising through children’s apps.
(3) Food and beverage products issued in children’s apps are not suitable for a healthy diet due to their low (or no) nutritional value.
(4) The number of non-compliant ads has been significantly higher than those that comply with all standards and recommendations.

The truth is that, in the light of the data extracted, we dare to say that despite the existence of a strong code, within a global strategy (NAOS), its application is not proving effective, probably due to the laxity of its practice, as well as the practical non-existence of sanctions that penalize the violation of the principles contemplated in the PAOS code.

On the other hand, food advertising content aimed at children not only skips the PAOS Code but also goes unnoticed by the target audience (consumers, kids, buyers, parents). And, in this way, the levels of remembrance and recognition are eminently high, thus provoking an increase in the probability of purchase intention and, consequently, of consumption, despite being food products whose ingredients and components are clearly identified as harmful or unhealthy by the World Health Organization.

This is why the media, in general, and mobile devices in particular (meaning mobile phones such as smartphones and tablets), must continue to work, hand in hand with educational, family and legislative entities, and without losing sight of the concept of social marketing within a happiness management, to channel their function towards a social good, as is being done with other media.

Therefore, since most food products are bought or ordered as a direct result of advertising and companies need to sell their products, and since many children want to be fashionable and try something new, we propose a balance from the field of education and communication, making all parties co-responsible, although with different charges in the responsibility. Thus, we must educate our children by making them critical of the media in general, of advertising and, more specifically, of unhealthy food advertising. Besides, parents should have more influence on this critical spirit, as well as they should control the contents to which minors are exposed.

Food companies need to sell their products, but they should make healthier products with less saturated fats and sugars. In this way, if food products were healthier, there would be no criticism of the fact that they try to sell their products, with or without advertising. If, this change is not done voluntarily by the advertisers, this is the time when the health authorities should intervene in a conscious and responsible manner. Advertisers and agencies should create joint strategies to develop educational food advertising, directing its influence towards healthy, balanced nutrition complemented by physical exercise. And public administrations should strengthen compliance with their laws and codes.

## Figures and Tables

**Table 1:** PAOS Code compliance and its ethical norms on mobile devices in Spain, 2019.

Code	Ethical Norms	Compliance		Not Compliance		Unclear	
		*n*	%	*n*	%	*n*	%
I	Principle of legality	34	46.57	4	5.48	35	
II	Principle of loyalty	73	100	-	-	-	-
III	Education and nutritional information	29	39.72	19	26.03	25	34.24
IV	Presentation of the products	9	12.33	39	53.42	25	34.25
V	Product information	5	6.84	40	54.79	28	38.36
VI	Sales pressure	23	31.51	29	39.72	21	28.77
VII	Support and promotion through characters and programs	68	93.15	5	6.84	-	-
VIII	Identification of advertising	73	100	-	-	-	-
IX	Comparative presentations	73	100	-	-	-	-
X	Promotions, raffles, contests and children’s clubs	34	46.57	39	53.42	-	-
XI	Safety	27	36.98	31	42.46	15	20.55
XII	Treatment of personal data	67	91.78	2	2.74	4	5.48
XIII	Viral marketing	69	94.52	-	-	4	5.48
XIV	Protection against inappropriate content	69	94.52	-	-	4	5.48

**Table 2:** Frequency of non-compliance with PAOS Code norms and ethical standards by food and beverage ads (F&B) aimed at children through mobile devices in Spain in 2019.

Ads	Ethical Norms	1	2	3			4					5	6				7		8	9	10				11		12					13	14		
	Ethical Standards	-	-	1	2	3	4	5	6	7	8	9	10	11	12	13	14	15	16	17	18	19	20	21	22	23	24	25	26	27	28	29	30	31	32
1	51	X										X										X	X		X	X									
2	9						X	X	X	X		X			X						X	X													
3	6			X								X													X	X									
4	18																																		
5	30			X									X	X																					
6	20						X	X		X					X		X				X	X	X		X	X									
7	9						X		X						X																				
8	23						X			X											X	X	X	X			X								
9	3								X	X					X																				
10	1							X				X					X																		

**Table 3:** PAOS Code compliance/not compliance.

N°	Brand	Level of Compliance
1	Ositos	Not in compliance
2	Babybel	Unclear
3	Fanta	Not in compliance
4	Danone	Unclear
5	Danonino	Not in compliance
6	La vaca que ríe	Unclear
7	Galletas Príncipe	Not in compliance
8	Chips Ahoy	Not in compliance
9	Choco Flakes	Not in compliance
10	Haribo	Not in compliance
11	(Caffeine free) Coca Cola	Not in compliance
12	Danup	Not in compliance
13	Telepizza	Not in compliance
14	Hero baby	Not in compliance
15	McDonalds	Unclear
16	Donuts	Not in compliance
17	Burger King	Not in compliance
18	Tosta Rica	Not in compliance
19	Puleva	Unclear
20	Oceanix	Not in compliance
21	Dinosaurus	Not in compliance
22	Sunny Delight	Not in compliance
